# Physico-Chemical Properties of Soybean Meal-Based Adhesives Reinforced by Ethylene Glycol Diglycidyl Ether and Modified Nanocrystalline Cellulose

**DOI:** 10.3390/polym9090463

**Published:** 2017-09-20

**Authors:** Xiaona Li, Mingsong Chen, Jizhi Zhang, Qiang Gao, Shifeng Zhang, Jianzhang Li

**Affiliations:** 1 Ministry of Education (MOE) Key Laboratory of Wooden Material Science and Application, Beijing Key Laboratory of Wood Science and Engineering, School of Materials Science and Technology, Beijing Forestry University, Beijing 100083, China; lixiaona510@bjfu.edu.cn (X.L.); chen_boss@bjfu.edu.cn (M.C.); gaoqiang@bjfu.edu.cn (Q.G.); 2 Key Laboratory for Liquid-Solid Structural Evolution and Processing of Materials (MOE), School of Materials Science and Engineering, Shandong University, Jinan 250061, China; zjzvip@sdu.edu.cn

**Keywords:** nanocrystalline cellulose, soybean meal-based adhesive, KH560, plywood

## Abstract

An eco-friendly soybean meal-based adhesive (SM adhesive) was developed by incorporating ethylene glycol diglycidyl ether (EGDE) and nanocrystalline cellulose (NCC). In order to introduce epoxy groups, NCC was modified by KH560 (denoted as MNCC). The functional groups, thermal stability, and cross section of the resultant adhesive were characterized. Three-ply plywood was fabricated to measure the dry and wet shear strength of the adhesive. The experimental results showed that the epoxy groups on MNCC reacted with the carboxyl group of SM protein molecules, forming a crosslinking network and a ductile adhesive layer. As a result, compared with the SM adhesive modified by EGDE, the thermal stability of the adhesive with MNCC was improved and the wet shear strength was increased to 1.08 MPa.

## 1. Introduction

For excellent bonding strength and water resistance, most present available commercial adhesives for wood industry are petroleum based, such as phenol formaldehyde (PF), urea formaldehyde (UF), melamine urea formaldehyde (MUF). Being a human carcinogen as well as an unsustainable and non-renewable resource, petroleum-based polymers and composites have caused considerable human health and environmental problems [1,2,3].

The soybean meal (SM) mainly consists of protein, and with the advantages of renewability, biocompatibility, biodegradability, processability, and film-forming capacity, it has great potential to be used in wood industry, agriculture, bioscience, and biotechnology [4,5,6]. Soy-based adhesive is produced from the soybean meal, a processing residue of soybean when producing soybean oil and other soy-based food products, which were commercially used in the production of plywood from the 1930s to the 1960s [7].

However, the panels bonded with these soy-based adhesives had poor water resistance, and so they were replaced by panels bonded with synthetic formaldehyde-based adhesive. The expanding wood adhesives market and environmental protection concerns have brought about close attention to and an urgent need for the development of environmentally friendly alternative wood adhesives based on renewable resources [1,8,9,10].

Abundant and renewable SM-based adhesive, with high biodegradability, has aroused further interest by many researchers [11,12,13]. Many efforts have been done to improve the performance of soy-based adhesives by modifying soy proteins with chemicals [8,14,15], resins [16,17], and enzymes [18], etc. However, the overall performance of these modified soy proteins, in terms of adhesive strength and water resistance, is still not comparable with synthetic resins.

Nanomaterials have shown excellent achievements in enhancing the performance of polymers and adhesives [19,20]. Among these nanomaterials, renewable nanocrystalline cellulose (NCC) was the most promising one, which can be obtained from natural fibers—such as wood and cotton—by removing lignin, hemicellulose, reducing the amorphous regions of the fibers, and breaking down the chain of the cellulose to nanoscale units [21,22,23]. NCC has a special rod-like structure and a great amount of hydroxyl groups on its surface, showing a great potential application in improving the performance of the matrix. NCC was used as a filler to enhance the fracture mechanical properties of adhesives and the results showed that the significant improvement in the fracture toughness of brittle polymers was achieved due to the fiber-bridging effects of cellulose nano-fibrils [24]. By incorporating 0.5 wt % of NCC into the SM adhesive, the bonding strength could increase by 20%. The explanation of the increase probably was that the incorporation of NCC promoted the curing process of the modified soy adhesives, resulting in fewer holes and cracks in the cured adhesives. The tight cured adhesive structure prevented the moisture intrusion, and thus improved the water resistance of the adhesives [25]. Undoubtedly, these interactions relied on the physical bonding between the adhesives and fibers. However, there is no denying that this physical enhancement is limited.

If the enhancement of soybean protein adhesives and cellulose materials can be achieved, like forming chemical bonding, the performance of soybean protein bonding composites will be significantly improved. An alternative approach uses surface modification to attach a functional group to the surface of NCC. Then the functional group on the surface of NCC react with the matrix, which significantly increases the performance of the composites.

## 2. Materials and Methods

### 2.1. Materials

SM (45.2% of soy protein, 5% of moisture) was purchased from Sanhe Hopefull Group Oil Grain Food Co. Ltd. Poplar (Populus tomentosa Carr.) veneers (400 mm × 400 mm × 1.5 mm, 8% of moisture) were obtained from Langfang, China. γ-glycidylpropyltrimethoxysilane (KH560) were purchased from Nanjing Xinhuai Scientific Co. Ltd. (Nanjing, China). NCC was purchased from the Beijing Nanocrystalline Cellulose Technology Research & Development Center (Beijing, China) with a diameter of 30–60 nm, length of 200–500 nm, and degree of crystallinity of 79.74%. The ethylene glycol diglycidyl ether (EGDG) was obtained from Chuzhou Huisheng Electronic Material Co., Ltd. (Chuzhou, China). Other chemicals were analytical grade and obtained from Beijing Chemical Reagents Co. (Beijing, China). All chemical reactants were analytical grades.

### 2.2. Modification of NCC (MNCC)

The silane coupling agent KH560 (20 g) was firstly hydrolyzed in the 1000 mL mixture of ethanol and water (90/10 by volume). The pH value of the solution was adjusted to 4–5 with acetic acid and stirred continuously for 4 h. Next, 25 g of NCC was added into the solution and refluxed at 70 °C with the magnetic stirring and in water bath for 24 h. Then the products were washed several times with a mixture of ethanol and water to remove the unreacted silane coupling agent and impurities. The obtained MNCC was finally dried at 90 °C to a constant weight.

### 2.3. Preparation of Modified SM-Based Adhesives

The following is a representative procedure for the preparation of the different soybean meal-based adhesives.

MSM (Modified Soymeal adhesive) Adhesive: the tap water (75 g), SM (25 g), and EGDE (6 g) were sequentially added to a three-neck flask and stirred uniformly at room temperature. Then ultrasonically dispersed for 3 min by VCX 800 of SONICS (Newtown, CT, USA).

MSM/NCC Adhesive: the tap water (75 g), SM (25 g), EGDE (6 g), and NCC (1 g) were sequentially added to a three-neck flask and stirred uniformly at room temperature. Then ultrasonically dispersed for 3 min by VCX 800 of SONICS (Newtown, CT, USA).

MSM/MNCC Adhesive: tap water (75 g), SF (25 g), EGDE (6 g), and MNCC (1 g) were sequentially added to a three-neck flask and stirred uniformly at room temperature. Then ultrasonically dispersed for 3 min by VCX 800 of SONICS (Newtown, CT, USA).

### 2.4. General Test Methods

In the FTIR test, the structural changes of various adhesives were examined using the Nicolet 6700 spectrometer (Nicolet Instrument Corporation, Madison, WI, USA) from 500 to 4000 cm^−1^ with a 4 cm^−1^ resolution with 32 scans using KBr tableting method.

In the TGA test, the thermal stability of the samples were evaluated by thermogravimetric analysis TGA (Q50, TA Instruments, New Castle, DE, USA) at a constant heating rate of 10 °C/min from room temperature to 600 °C under a flowing nitrogen atmosphere.

In the SEM-EDS test, the cured adhesive films with a thickness of about 1.5 mm were fractured and the fracture surface of the cured adhesives was observed by the Hitachi S-3400 N (Hitachi Science System, Ibaraki, Japan) scanning electron. Before the SEM-EDS test, the fractured surface was sputter-coated with gold. In addition, the energy dispersive X-ray spectroscopy (EDS, EX-350, Horiba Scientific, Fukuoka, Japan) was utilized for the elemental analysis.

### 2.5. Preparation of Three-Ply Plywood Samples

Poplar veneers with a dimension of 400 mm × 400 mm × 1.5 mm were used. The SM-based adhesive was coated on both sides of the core veneer using a brush. The dosage of adhesive was maintained about 185 g/m^2^. The adhesive-coated veneer was stacked between two uncoated veneers with the grain direction of the two adjacent veneers perpendicular to each other. These stacked veneers were hot-pressed at 1.0 MPa, 120 °C for 6 min. After the hot-pressing, the panels were stored at ambient temperature for at least 24 h prior to be cut into specimens for the evaluation of the shear strength and water resistance.

### 2.6. Shear Strength and Water Resistance

The shear strength of plywood was determined according to the Chinese National Standard for Type II plywood (GB/T 17657-2013). Three-ply poplar plywood was prepared at a 120 °C hot pressing temperature with a 1.0 MPa pressure. The panels bonded with the different soybean meal-based adhesives were equilibrated for at least 24 h under room conditions and cut into specimens with a dimension of 100 mm × 25 mm (glued area of 25 mm × 25 mm). The dry shear strength was determined using a common tensile machine operating at a speed of 10.0 mm/min. The force required to break the glued specimen was recorded. The shear strength was calculated as the ratio of the force/glue area. The reported strength data were averaged over six specimens. Meanwhile, the cut wood specimens were immersed in water at 63 ± 3°C for 3 h, removed from water and cooled at room temperature for 10 min prior to the measurements. The wet shear strength of the wood specimens was recorded under the same testing condition. The reported strength data were the averages of six specimens. Wood failure of samples after shear strength testing were determined based on the area method. The glue interface was broken after shear strength testing. Part of wood the tissue was torn from one veneer and bonded on another veneer. The ratio of the torn area/glue area represents the wood failure.

## 3. Results and Discussion

### 3.1. The Modification of the NCC

**Figure 1:**
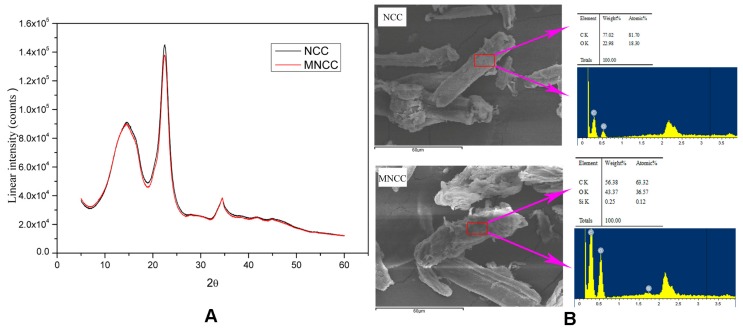
X-ray diffraction patterns (**A**) and EDS analysis (**B**) of NCC and MNCC.

**Scheme 1:**
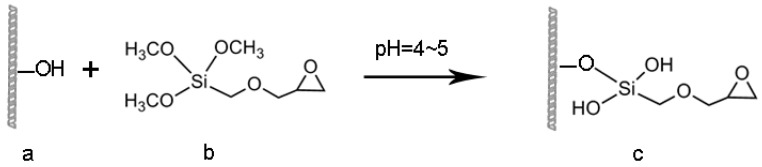
Schematic illustration of NCC modified by silane coupling agent: (**a**) NCC; (**b**) KH-560; (**c**) MNCC.

X-ray diffractometry was used to evaluate the modification effect of NCC (Figure 1). The diffractogram profiles of the samples, show the characteristic of type I cellulose indicated by the presence of the peaks at 2θ = 14.5° (plane 101), 22.5° (plane 002), and 34.5° (plane 004) respectively [26,27]. Figure 1 shows the MNCC still maintained the crystalline structure the same as NCC. However, the modification can prevent agglomeration of fibers, resulting in a weak diffraction peak [28]. It can be clearly seen that the crystallization peak intensity of MNCC decreased in comparison to the natural NCC, indicating that important reactions had occurred on the NCC surfaces. The EDS analysis of Si content in MNCC is shown in Figure 1B. The presence of Si elements was confirmed at about 0.25% weight percent. The above analysis proved that NCC was successfully modified by silane coupling agent. The reaction between silane coupling agent and NCC is illustrated in Scheme 1.

### 3.2. FTIR Analysis

**Figure 2:**
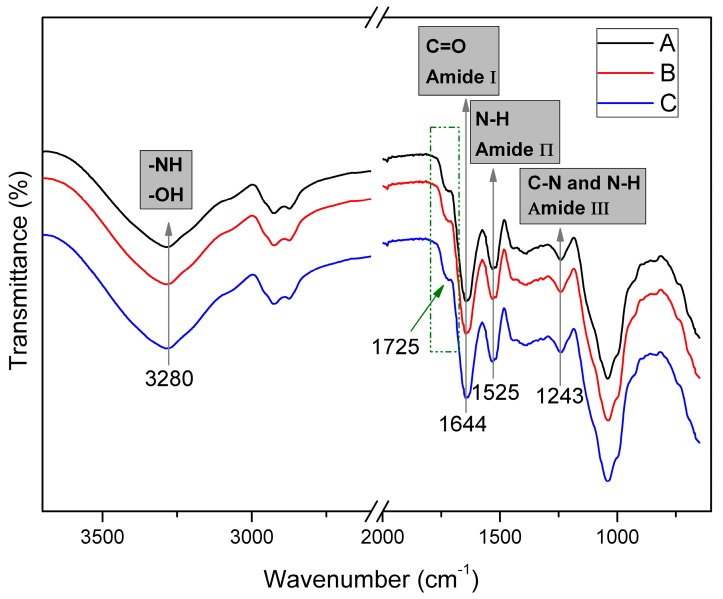
FTIR spectra of soybean meal-based adhesives: (**A**) MSM adhesive, (**B**) MSM/NCC adhesive, and (**C**) MSM/MNCC adhesive.

**Scheme 2:**
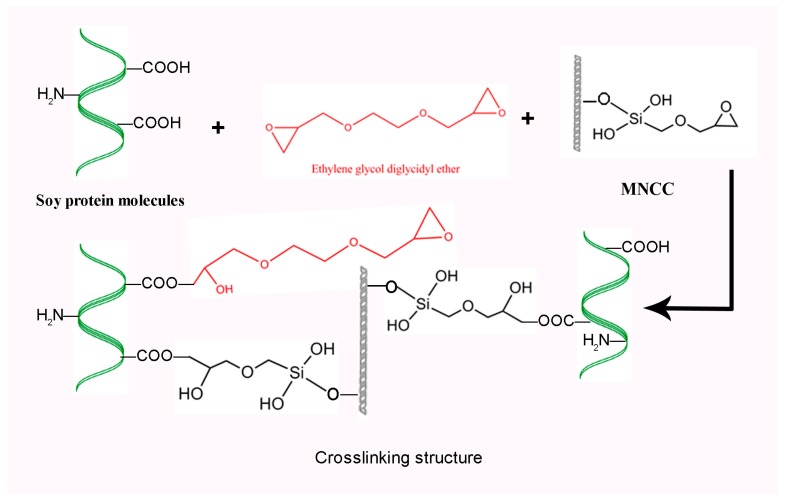
Schematic illustration of the reaction mechanism of EGDE, MNCC, and soy protein molecules.

FTIR examination was carried out to reveal the possible chemical and physical interactions of the SM-based adhesive. However, compared with the spectra of MSM adhesive, the FTIR analysis did not detect a new IR absorption peak after introducing NCC and MNCC to the MSM adhesive but rather increased peak intensity at approximately 3280 cm^−1^ (attributed to the stretching mode of –NH and –OH groups), which was ascribed to the stretching vibration of hydroxyl groups in NCC and MNCC. All spectra exhibit the relevant peaks at 1644, 1525, and 1243 cm^−1^ (Figure 2), which are characteristic of amide I (C=O stretching), amide II (N–H bending), and amide III (C–N and N–H stretching), respectively [29,30]. Interestingly, there was a new small peak that appeared near 1725 cm^−1^, not belonging to SM, which was related to the carbonyl group of the ester bond and originated from the esterification reaction between epoxy groups and carbonyl groups of the soy protein [31]. The above analysis indicated that NCC and MNCC successfully incorporated into the adhesive, and the epoxy group of EGDE and MNCC reacted with SM protein molecules. The crosslinking reaction generated a more stable chemical bond and formed a crosslinking network, which enhanced the performance of the SM-based adhesive. The reaction mechanisms of EGDE, MNCC, and soy protein molecules are shown in Scheme 2.

### 3.3. Thermal Stability Analysis of SM-Based Adhesive

**Figure 3:**
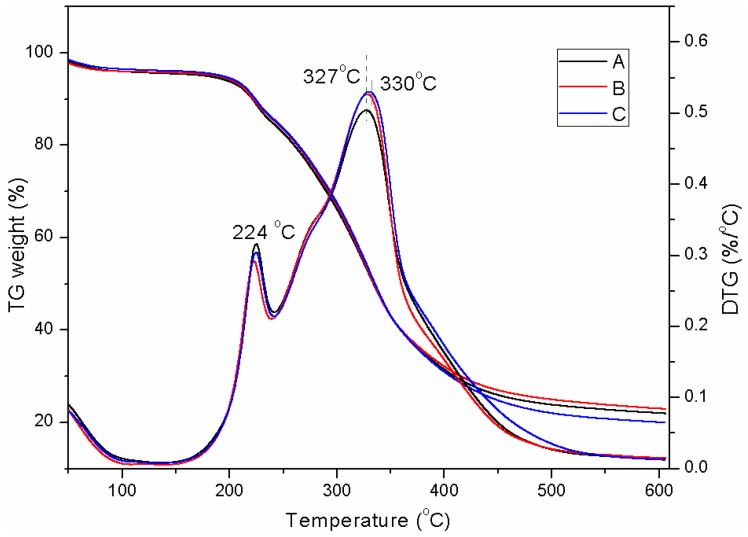
The TG and DTG curves of the adhesive: (**A**) MSM adhesive, (**B**) MSM/NCC adhesive, and (**C**) MSM/MNCC.

Thermo-gravimetric analysis is an effective method for analyzing the thermal stability of a material. Figure 3 presents two obvious degradation peaks. The first peak appeared near 224 °C was attributed to the elimination of water in the initial stages up to 100 °C and the dissociation of the quaternary structure of the protein in the later stages [32]. For all SM-based adhesives, the first degradation peak temperature was the same (224 °C). The second peak, between 250 and 400 °C was primarily due to the breakage of the covalent peptide bonds in the amino acid residues. For the MSM and MSM/NCC adhesives, the second stage breakage temperature was the same (327 °C). In the system of MSM/NCC, the physical filling occurred between NCC and the SM protein molecules [33], which had little effect on the thermal stability of the resultant adhesive. As a result, adding NCC did not improve the thermal stability of the resultant adhesive. Differently, the addition of MNCC, the second stage breakage temperature of the MSM/MNCC adhesive slightly increased from 327 to 330 °C. Generally, crosslinking will increase the thermal stability of the resultant adhesive due to the formation of chemical bonds [34]. The epoxy groups on the surface of NCC reacted with the SM protein molecules as described in the FTIR analysis, which formed a more stable structure/crosslinking network (as showed in Scheme 2). Therefore, incorporation of MNCC increased the thermal stability of the resultant adhesive.

### 3.4. SEM Analysis

**Figure 4:**
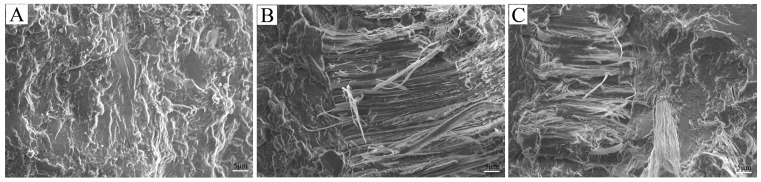
Cross-sectional scanning electron microscopy photographs of the adhesive: (**A**) MSM adhesive, (**B**) MSM/NCC adhesive, and (**C**) MSM/MNCC adhesive.

Generally, crosslinking is conducive to forming a density cross section [35,36]. As the analysis of FTIR, the epoxy groups of EGDE reacted with SM protein molecules to form a crosslinking structure, resulting in a homogenous cross section of the cured MSM adhesive without cracks or holes (Figure 4A). After adding NCC, instead of breaking the homogenous structure, the cross section became slightly rough, which implied that the physical filling effect of NCC did not break the crosslinking structure of the MSM adhesive. This result implied that the presence of NCC could reduce the brittleness of the composites [37]. Furthermore, the fractured surface became rougher (Figure 4C) after incorporating MNCC, exhibiting somewhat ductile fracture property. The formation of the ductile fracture probably resulted from the chemical crosslinking reaction among SM protein molecules, MNCC, and EGDE in the matrix. These results were consistent with our previous study [38]. When being impacted by an external force, the ductile structure of the cured adhesive layer could effectively resist the propagation of layer cracks. As a result, the water resistance of the cured adhesive is therefore enhanced.

### 3.5. Dry Strength and Wet Shear Strength Measurement

Typically, crosslinking makes a network that potentially improves the compactness and water resistance of the cured adhesive layers [3,35]. The dry strength is shown in Table 1. For the MSM adhesive, the dry strength of the resultant plywood was 1.30 MPa with a low wood failure (5–10%). After adding NCC and MNCC, the dry strength increased to 1.38 and 1.84 MPa respectively. Differently, the plywood bonded by MSM/MNCC adhesive showed a 100% wood failure, indicating that MNCC effectively improved the dry strength of the specimens.

**Figure 5:**
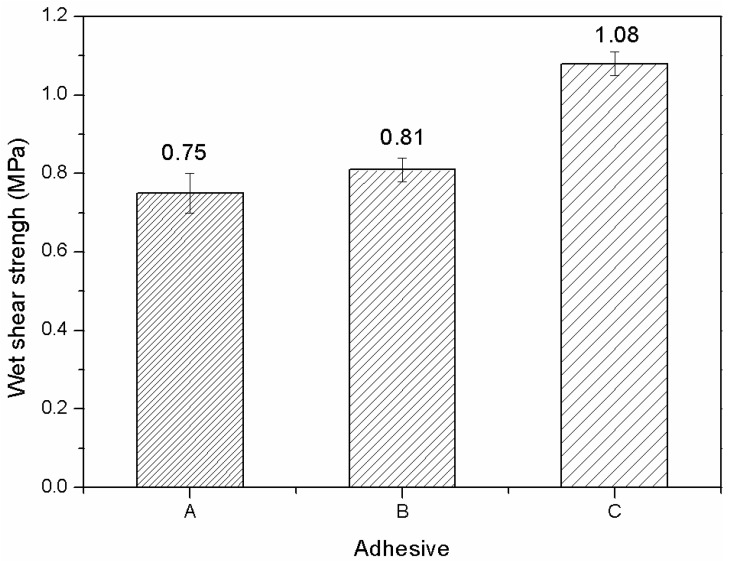
The wet shear strength of the adhesive: (**A**) MSM adhesive, (**B**) MSM/NCC adhesive, and (**C**) MSM/MNCC adhesive.

The wet shear strength of the plywood bonded by different adhesives is shown in Figure 5. The wet shear strength of the plywood bonded with MSF adhesive was improved to 0.75 MPa, compared with our previous studies (the wet shear strength of the plywood bonded by the soy meal only adhesive was about 0.3–0.4 MPa) [35,39], which was attributed to the crosslink action that occurred between the EGDE and the SM protein molecules [38]. The wet shear strength of the plywood bonded by the MSM adhesive (0.75 MPa) can meet the interior use plywood requirement (≥0.7 MPa) according to China National Standard (GB/T 9846.3-2013). However, for factory production, this strength is not enough. The addition of NCC, the wet shear strength was increased to 0.81 MPa, because of the physical filling effect between NCC and the MSF adhesive [33]. As the analysis of FTIR and SEM, the epoxy groups on the MNCC surfaces reacted with SM protein molecules, forming a crosslinking network and ductile cured adhesive layer. As expected, the introducing of MNCC to the MSF adhesive, the wet shear strength was improved to 1.08 MPa counting for an increase of 44%.

## 4. Conclusions

NCC was successfully modified by silane coupling agent (KH560), that is to say, epoxy groups were effectively introduced to the NCC surfaces. The epoxy groups of MNCC and EGDE reacted with the SM protein molecules, forming a chemical crosslinking and ductile structure. As a result, the thermal stability, dry strength, and wet shear strength were improved. The work showed the potential to use nanoparticle NCC for improving the physicochemical properties of SM protein-based adhesives.

## Figures and Tables

**Table 1:** Dry strength and wood failure of plywood bonded by the soybean meal-based adhesives.

Adhesives	Dry strength (MPa)	Wood failure (%)
A (MSM adhesive)	1.30 ± 0.12	5–10
B (MSM/NCC adhesive)	1.38 ± 0.14	10–20
C (MSM/MNCC adhesive)	1.84 ± 0.23	100

## References

[1] Pizzi A. (2006). Recent developments in eco-efficient bio-based adhesives for wood bonding: Opportunities and issues. J. Adhes. Sci. Technol..

[2] Tejado A., Pena C. (2007). Physico-chemical characterization of lignins from different sources for use in phenol–formaldehyde resin synthesis. Bioresour. Technol..

[3] Wu Z., Xi X. (2017). Soy-based adhesive cross-linked by phenol-formaldehyde-glutaraldehyde. Polymers.

[4] Khan M.K., Schutyser M.A. (2012). The potential of electrospraying for hydrophobic film coating on foods. J. Food Eng..

[5] Frihart C.R., Coolidge T. (2016). High bonding temperatures greatly improve soy adhesive wet strength. Polymers.

[6] Song F., Tang D.L. (2011). Biodegradable soy protein isolate-based materials: A review. Biomacromolecules.

[7] Huang J., Gu K. (2013). Development and evaluation of new curing agents derived from glycerol for formaldehyde-free soy-based adhesives in wood composites. Holzforschung.

[8] He Z. (2017). Bio-Based Wood Adhesives: Preparation, Characterization, and Testing.

[9] Nordqvist P., Lawther M. (2012). Adhesive properties of wheat gluten after enzymatic hydrolysis or heat treatment—A comparative study. Ind. Crop. Prod..

[10] Deka H., Wang T. (2015). Novel biocomposites from biobased epoxy and corn-based distillers dried grains (DDG). J. Poly. Environ..

[11] Nishinari K., Fang Y. (2014). Soy proteins: A review on composition, aggregation and emulsification. Food Hydrocoll..

[12] Cui Z., Kong X. (2014). Effects of rutin incorporation on the physical and oxidative stability of soy protein-stabilized emulsions. Food Hydrocoll..

[13] Cheng H., Ford C. (2016). Soy and cottonseed protein blends as wood adhesives. Ind. Crop. Prod..

[14] Qi G., Li N. (2013). Physicochemical properties of soy protein adhesives modified by 2-octen-1-ylsuccinic anhydride. Ind. Crop. Prod..

[15] Jang Y., Huang J. (2011). A new formaldehyde-free wood adhesive from renewable materials. Int. J. Adhes. Adhes..

[16] Gao Q., Shi S. (2011). Improved plywood strength and lowered emissions from soybean meal/melamine urea-formaldehyde adhesives. For. Prod. J..

[17] Fan D., Qin T. (2011). A soy flour-based adhesive reinforced by low addition of MUF resin. J. Adhes. Sci. Technol..

[18] Kumar R., Choudhary V. (2004). Enzymatically-modified soy protein part 2: Adhesion behaviour. J. Adhes. Sci. Technol..

[19] Liang K., Shi S. (2011). Nanoclay filled soy-based polyurethane foam. J. Appl. Polym. Sci..

[20] Shi J., Shi S. (2011). Kenaf bast fibers—Part II: Inorganic nanoparticle impregnation for polymer composites. Int. J. Polym. Sci..

[21] Zhou Q., Malm E. (2009). Nanostructured biocomposites based on bacterial cellulosic nanofibers compartmentalized by a soft hydroxyethylcellulose matrix coating. Soft Matter..

[22] Alexandrescu L., Syverud K., Belosi F. (2012). Nanofibers against nanoparticles: Cellulosic nanofibers for nanoparticle aerosol filtration, concepts, achievements, and perspectives. Abstracts of Papers of the American Chemical Society.

[23] Cranston E., Gray D. (2006). Morphological and optical characterization of polyelectrolyte multilayers incorporating nanocrystalline cellulose. Biomacromolecules.

[24] Khalili S., Jafarkarimi M. (2009). Creep analysis of fibre reinforced adhesives in single lap joints—Experimental study. Int. J. Adhes. Adhes..

[25] Gao Q., Li J. (2012). Soybean meal-based adhesive reinforced with cellulose nano-whiskers. Bioresources.

[26] Kalita E., Nath B. (2015). High quality fluorescent cellulose nanofibers from endemic rice husk: Isolation and characterization. Carbohydr. Polym..

[27] Santos R., Neto W. (2013). Cellulose nanocrystals from pineapple leaf, a new approach for the reuse of this agro-waste. Ind. Crop. Prod..

[28] Yan Y., Amer H. (2016). Dry, hydrophobic microfibrillated cellulose powder obtained in a simple procedure using alkyl ketene dimer. Cellulose.

[29] Gao Z., Zhang Y. (2015). The effects of thermal-acid treatment and crosslinking on the water resistance of soybean protein. Ind. Crop. Prod..

[30] Ghosh D., Netravali A. (2013). A soy flour based thermoset resin without the use of any external crosslinker. Green Chem..

[31] Chu F., McKenna T. (1997). A study of the preparation and mechanism of the ambient temperature curing of acrylic latex with epoxy resins. Polymer.

[32] Swain S., Rao K. (2005). Biodegradable polymers: IV. Spectral, thermal, and mechanical properties of cross-linked soy protein concentrate. Polym. Int..

[33] Li H., Li C. (2014). Properties of soybean-flour-based adhesives enhanced by attapulgite and glycerol polyglycidyl ether. Ind. Crop. Prod..

[34] Swain S., Rao K. (2005). Biodegradable polymers; Part II. Thermal degradation of biodegradable plastics cross-linked from formaldehyde-soy protein concentrate. J. Therm. Anal. Calorim..

[35] Li J., Luo J. (2015). Soybean meal-based wood adhesive enhanced by ethylene glycol diglycidyl ether and diethylenetriamine. Ind. Crop. Prod..

[36] Luo J., Li X. (2016). Effects of polyisocyanate on properties and pot life of epoxy resin cross-linked soybean meal-based bioadhesive. Appl. Polym..

[37] Barari B., Omrani E. (2016). Mechanical, physical and tribological characterization of nano-cellulose fibers reinforced bio-epoxy composites: An attempt to fabricate and scale the ‘Green’ composite. Carbohydr. Polym..

[38] Zhang S., Xia C. (2016). Soy protein isolate-based films reinforced by surface modified cellulose nanocrystal. Ind. Crop. Prod..

[39] Li X., Li J. (2016). A SEP united crosslinked network in a SM wood adhesive and its performance. RSC Adv..

